# DV-DETR: Improved UAV Aerial Small Target Detection Algorithm Based on RT-DETR

**DOI:** 10.3390/s24227376

**Published:** 2024-11-19

**Authors:** Xiaolong Wei, Ling Yin, Liangliang Zhang, Fei Wu

**Affiliations:** School of Electronic and Electrical Engineering, Shanghai University of Engineering Science, Shanghai 201620, China; m320122403@sues.edu.cn (X.W.); zhangliang061@foxmail.com (L.Z.); fei_wu1@163.com (F.W.)

**Keywords:** transformer, small target detection, real-time task, RT-DETR algorithm

## Abstract

For drone-based detection tasks, accurately identifying small-scale targets like people, bicycles, and pedestrians remains a key challenge. In this paper, we propose DV-DETR, an improved detection model based on the Real-Time Detection Transformer (RT-DETR), specifically optimized for small target detection in high-density scenes. To achieve this, we introduce three main enhancements: (1) ResNet18 as the backbone network to improve feature extraction and reduce model complexity; (2) the integration of recalibration attention units and deformable attention mechanisms in the neck network to enhance multi-scale feature fusion and improve localization accuracy; and (3) the use of the Focaler-IoU loss function to better handle the imbalanced distribution of target scales and focus on challenging samples. Experimental results on the VisDrone2019 dataset show that DV-DETR achieves an mAP@0.5 of 50.1%, a 1.7% improvement over the baseline model, while increasing detection speed from 75 FPS to 90 FPS, meeting real-time processing requirements. These improvements not only enhance the model’s accuracy and efficiency but also provide practical significance in complex, high-density urban environments, supporting real-world applications in UAV-based surveillance and monitoring tasks.

## 1. Introduction

With the rapid advancement of drone technology and significant cost reduction, drones have been widely used in various fields such as urban planning, traffic monitoring, agriculture, and environmental protection. Drone aerial photography has received great attention due to its ability to provide unprecedented low-cost high-resolution images. Meanwhile, real-time object detection algorithms, as an important research field in computer vision, have become widely applied in drone aerial photography [1]. However, the data captured by drones often contain a large number of small-sized targets, and due to limitations in flight altitude and complex terrain, object detection tasks face many challenges, such as small target size and severe occlusion. These factors greatly constrain the effective parsing of drone image data, thereby limiting the efficiency and practicality of drone technology in specific fields.

In recent years, deep learning, especially convolutional neural networks, has made significant progress in the field of image processing. By utilizing deep networks to automatically learn complex feature representations, deep learning methods perform well on traditional object detection tasks. Object detection algorithms are mainly divided into two categories [2]: two-stage detection algorithms based on candidate regions and single-stage detection algorithms based on direct prediction. Two-stage algorithms such as R-CNN [3] and its variants (Fast R-CNN, Faster R-CNN) first generate region candidates, and then perform classification and bounding box regression on these regions. This type of method usually has high detection accuracy, but high computational cost and poor real-time performance, making it difficult to meet the real-time processing needs of drones. Single-stage detection algorithms such as the YOLO (you only look once) [4,5,6,7,8] series directly predict the target category and position on the image through one forward propagation. Although it significantly improves processing speed compared to two-stage algorithms, YOLO’s speed and accuracy are negatively affected by NMS, which essentially does not achieve an end-to-end detection process. With the Transformer model becoming the standard paradigm in the field of natural language processing, the academic community has begun to explore its application in object detection tasks. In this context, the DETR [9,10] method serves as a groundbreaking example that reconstructs object detection as a sequence prediction problem. This method adopts the encoder–decoder structure of Transformer and combines a matching strategy based on a bipartite graph. Compared with traditional object detection techniques, DETR omits a large number of candidate box generation and non-maximum suppression processes, achieving direct correspondence between prediction results and image results. However, due to DETR’s adoption of a Transformer architecture with a large number of parameters, its real-time detection performance is limited when dealing with complex tasks. Zhao et al. [11] proposed an improved real-time detection version called RT-DETR to address the high computational cost issue of the DETR method. By optimizing computational efficiency, RT-DETR can achieve high training accuracy in fewer iterations. However, although RT-DETR has made significant progress in training efficiency, its encoding network still exhibits low efficiency in multi-scale feature recognition, and the fusion effect of different hierarchical features also needs to be improved. These limitations indicate that although RT-DETR provides innovation in real-time detection, further optimization is still needed in feature processing and fusion techniques. The GD-based scattering power decomposition method [12], by addressing pseudo-power components in complex backgrounds, can reduce background interference in polarimetric SAR data. This preprocessing can be particularly beneficial for small object detection in RT-DETR, enabling the model to better distinguish targets in challenging environments, such as dense vegetation and mixed-use landscapes.

In response to the above issues, and considering the current solutions for processing unmanned aerial vehicle images with high viewing angles and high dynamic backgrounds, which often suffer from insufficient detection accuracy due to insignificant small target features and easy loss, we improve and design a target detection model under unmanned aerial vehicle viewing angles based on the RT-DETR baseline—DV-DETR, and choose ResNet18 [13] as the backbone network to reduce the complexity of the model; we propose to use recalibration attention units [14] and reparameterization modules [15] to improve the baseline hybrid encoder, effectively capturing the complex dependency relationships between small targets and enhancing the model’s detection ability for small-sized targets. In addition, a variable attention mechanism [16] is adopted to replace the baseline AIFI module in order to better aggregate the features that need attention. On the bounding box regression loss function, Focaler-IoU [17] is used to focus on different regression samples, and the detection ability of the model for small-sized targets is enhanced by adjusting the weights of positive and negative samples. Through the above improved design, we expect that the model can improve the detection accuracy of small targets while ensuring real-time performance. Finally, the performance of the proposed DV-DETR model was validated through experiments.

## 2. Related Work

### 2.1. Challenges of YOLO Algorithm in Small Target Recognition

The YOLO algorithm [4,5,6,18] is an efficient single-stage object detection algorithm that achieves object classification and localization during a single image viewing process. This algorithm adjusts the size of the input feature map uniformly and uses grid partitioning to segment the image, ultimately generating bounding box coordinates and category probabilities. After continuous iteration and improvement, YOLO has developed into an important tool for real-time object detection and demonstrated superior performance in multiple benchmark tests, especially in terms of processing speed, surpassing many other single-stage detectors. However, in the task of detecting small targets, YOLO series algorithms face some unique challenges. Especially when using NMS to handle duplicate detection problems, although NMS effectively solves the problem of overlapping multiple detection boxes, it not only increases inference time but also introduces hyperparameters that may affect detection stability, which is ineffective in some complex scenarios. For example, in dense crowd detection scenarios, due to extremely close or even overlapping individual distances, NMS may mistakenly delete multiple valid detection results, resulting in a significant decrease in recall rate, which means that many correct targets are not detected. Due to the YOLO algorithm’s design preference for fast processing at the expense of accuracy, its sensitivity to small targets is relatively low. Although the YOLO series has significant advantages in speed and processing capability, its recall and accuracy in handling high-density small target scenes such as drone aerial photography still need to be improved.

### 2.2. End-to-End Object Detection Model

In response to the adverse effects and challenges of the NMS method on small target detection mentioned above, the RT-DETR model [11] can be used to quickly process multi-scale features. By utilizing its efficient hybrid encoder to decouple intra-scale interactions and cross-scale fusion of features at different scales, the inference speed has been improved. RT-DETR transforms the detection problem into an unordered sequence problem, transforming the originally dense detection into sparse detection. Therefore, this method can avoid the delay caused by non-maximum suppression NMS in YOLO series detectors [4], as long as the confidence ranking of feature classification scores is performed on the final prediction results, thereby improving detection speed. In terms of detection accuracy, the RT-DETR model introduces an uncertainty minimization query selection mechanism to improve detection accuracy by optimizing the initial selection of queries. This method selects the query that is most likely to represent the real object by evaluating the uncertainty of encoder features (i.e., the uncertainty of localization and classification), thereby reducing the uncertainty of the model in the decoding stage.

Although the RT-DETR model outperforms existing real-time detectors and similar-sized end-to-end detectors in terms of speed and accuracy, it has certain limitations. Especially in small target detection, the performance is still not as good as that of strong real-time detectors. Therefore, an improvement is needed to enhance the accuracy of small target detection while ensuring that the detection speed is not reduced too much.

## 3. Methods

### 3.1. RT-DETR Model

RT-DETR is a real-time end-to-end object detection model based on Transformer, which effectively processes multi-scale features by decoupling intra-scale interactions and cross-scale feature fusion, significantly reducing the computational cost of traditional DETR models. In terms of achieving faster processing speed and higher accuracy, RT-DETR is significantly superior to similar advanced YOLO models. The RT-DETR network mainly consists of three parts: the backbone network, the neck coding network, and the decoding prediction network. In the backbone network section, we selected mainstream ResNet18, SwinTransformer, and the baseline model’s backbone network HGNet for comparative experiments. The training results are shown in Table 1, where ResNet18 has a parameter count of 17.657M and FLOPs of 53.4G, making it the model with the lowest requirements among all candidate models, highlighting its practicality as a lightweight and efficient architecture. This makes ResNet18 particularly suitable for deployment in environments with limited computing resources. Although its mean average accuracy (mAP) is 45.407%, slightly lower than the other two models evaluated, the difference is still small and within an acceptable range. Therefore, in this study, ResNet18, which balances network depth and detection accuracy, was selected as the backbone network of the model to meet the specific requirements of small target detection in drone aerial photography.

In the neck coding network section, an efficient hybrid encoder was designed for the baseline model, which mainly consists of two modules: the Attention-Based Intra-scale Feature Interaction (AIFI) module based on the attention mechanism and the CNN-Based Cross-Scale Feature Fusion Module (CCFM) based on CNN. Although the proposed efficient hybrid encoder can enhance the detection ability of complex objects and scenes by iteratively fusing features from different levels, and gradually refine feature expression through iteration to improve detection accuracy and stability, this design also has certain limitations. Firstly, there may be insufficient fusion when dealing with multi-scale features, as it mainly focuses on improving processing speed, which may sacrifice the comprehensive effect of some features, especially in the multi-scale environment of drone aerial images where feature details may not be rich enough. This is one of the reasons why the model’s performance on small objects is still not good enough, as mentioned by the author. Secondly, AIFI relies on high-level feature extraction and cannot effectively utilize the local details and spatial information of low-level features. This article will further improve the neck network proposed for the baseline model.

### 3.2. Recalibrate Attention Unit

**Figure 1:**
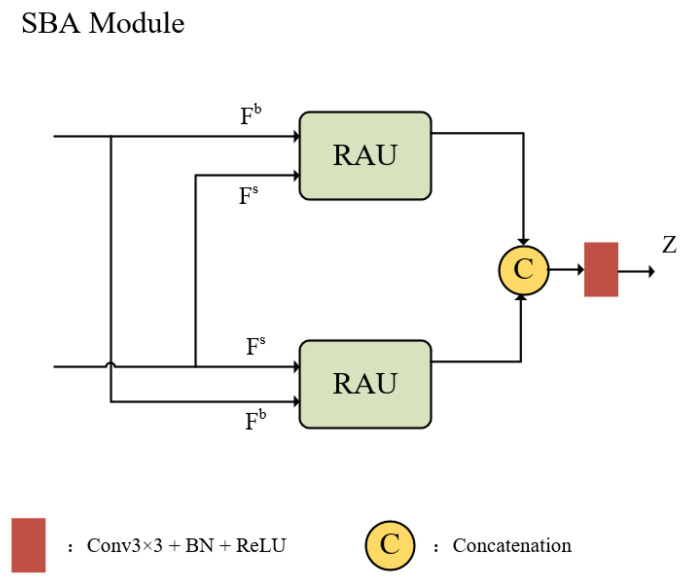
SBA module structure.

To address the challenge of detecting small targets, like vehicles and pedestrians, in drone aerial imagery, the recalibration attention unit (RAU) is introduced within the SBA module. In such images, small targets tend to be concentrated in lower and middle feature layers due to their limited size and feature detail. Traditional feature pyramid networks, with their top-down approach, often experience information loss, particularly when merging features across levels. Inspired by [14], we incorporated the RAU in our network to enhance feature retention by utilizing bidirectional fusion between high- and low-resolution features. This module, shown in Figure 1, better supports the detection of multi-scale targets by preserving crucial boundary and semantic information, thus improving object contours and location accuracy, especially in dense urban scenes where fine detail is critical.

**Figure 2:**
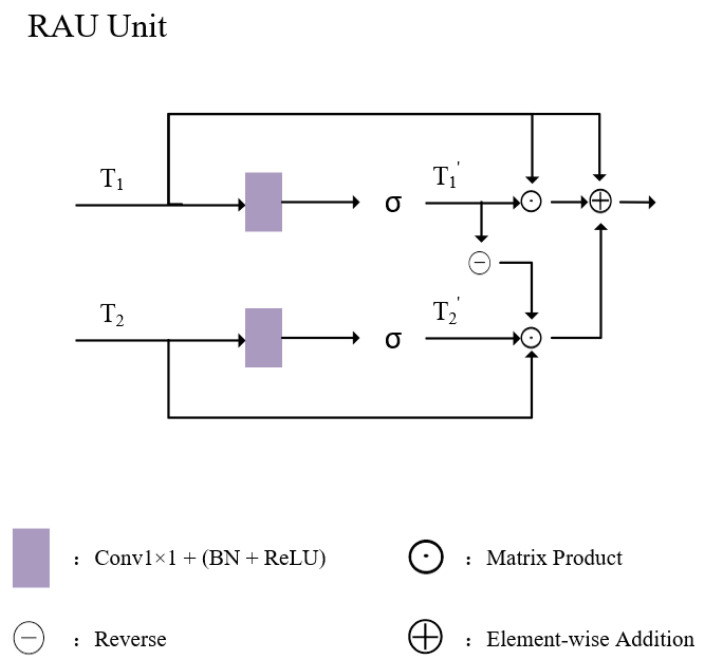
RAU unit structure.

Specifically, in order to fuse different hierarchical features more finely, the RAU adaptively extracts complementary information representations from two input features ($F^{s}$,$F^{b}$) before feature fusion. These two features come from the deep semantic information of the encoder and the shallow boundary detail features from the backbone network, respectively. As illustrated in Figure 2, both high-level and low-level information undergo different RAU processing methods to address the limitations of high-level features missing boundary details and low-level features missing semantic context.

To combine these enhanced features, outputs from both RAU units pass through a 3 × 3 convolution. The RAU function can be formulated as follows:

(1) $$T_{1}^{\prime }=W_{\theta }\left(T_{1}\right),T_{2}^{\prime }=W_{\phi }\left(T_{2}\right)$$

(2) $$PAU\left(T_{1},T_{2}\right)=T_{1}^{\prime }⊙T_{1}+T_{2}^{\prime }⊙T_{2}⊙\left(⊖\left(T_{1}^{\prime }\right)\right)+T_{1}$$

In this expression, $T_{1}$ and $T_{2}$ are input features processed through linear mappings $W_{\theta }\left(\cdot \right)$ and $W_{\phi }\left(\cdot \right)$, which use a 1 × 1 convolution to reduce the channel dimension to 32, producing feature maps $T_{1}^{\prime }$ and $T_{2}^{\prime }$. The operator ⊙ represents element-wise multiplication, while ⊖(·) denotes the complement of $T_{1}^{\prime }$.

The SBA module process can then be summarized as follows:

(3) $$Z=C_{3\times 3}(\mathrm{Concat}(P\mathrm{AU}(F_{s},F_{b}),P\mathrm{AU}(F_{b},F_{s})))$$

Here, $C_{3\times 3}\left(\cdot \right)$ is a 3 × 3 convolution followed by batch normalization and a ReLU activation layer. $F^{s}$, containing encoder-generated deep semantic details, is in $R^{\frac{H}{8}\times \frac{H}{8}\times 32}$, while $F^{b}$, carrying boundary information from the backbone, is in $R^{\frac{H}{4}\times \frac{H}{4}\times 32}$. Concat(·) concatenates along the channel dimension, resulting in the final output $Z\in R^{\frac{H}{4}\times \frac{H}{4}\times 32}$ for the SBA module.

### 3.3. Reparameterization Module

Multi-branch structures, such as ResNet [13], significantly enhance the extraction of multi-scale features by processing data streams at different scales in parallel, thus achieving better performance than single-branch structures in object detection tasks. However, this structure brings significant computational and parameter burdens due to its diverse parallel branches. To address this challenge, this study is inspired by the RepVGG network [15] and adopts a structural reparameterization strategy. This strategy uses a multi-branch structure in the training stage to optimize feature extraction and obtain better weight parameters, while in the inference stage, it switches to a single-branch structure to improve detection efficiency. Especially in object detection tasks from the perspective of drones, the similarity in features between tricycles, awning-tricycles, and motorcycles can easily lead to false positives. The use of the above-mentioned multi-parameter multi-branch structure can effectively extract multi-scale features, thereby solving such false detection problems, while the reparameterization process ensures the efficiency of the inference stage.

**Figure 3:**
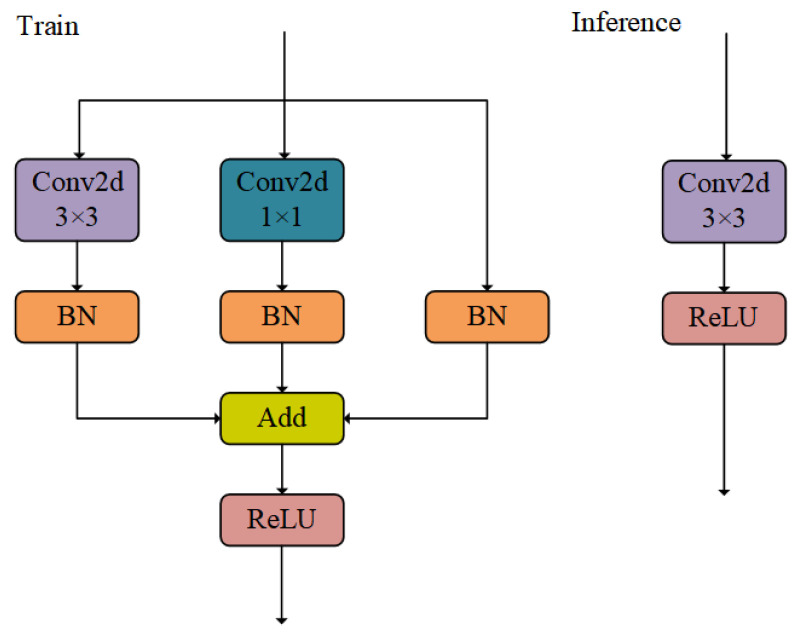
RepConv module.

We improve the backbone network of the model by introducing the RepConv module to replace traditional convolution, in order to enhance the fusion and extraction of multi-scale features. The module structure is shown in Figure 3. As the core of the RepVGG network, the RepConv module enriches multi-scale information through batch normalization (BN) and parallel structures of 1 × 1 and 3 × 3 convolutions during the training phase. In the validation phase, the multi-branch structure is fused into a single branch through reparameterization techniques, achieving efficient and high-precision detection of the model.

### 3.4. Deformable Attention Mechanism

In drone aerial image detection, scenes are often complex and variable, posing challenges for static feature fusion methods. The efficient hybrid encoder in the RT-DETR baseline model adopts a static feature fusion strategy, which may appear less flexible when dealing with complex and changing scenes compared to attention mechanisms that can dynamically adjust attention points. Inspired by [16], we introduce the deformable attention mechanism called DAttention. Unlike the traditional ViT [19] model, this mechanism allows for adaptive focus on relevant regions in the feature map, guided by sampling points generated through an offset network, thereby enhancing the ability to capture relationships among objects.

**Figure 4:**
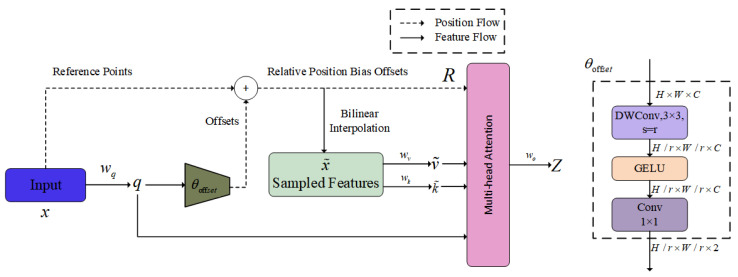
Deformable attention structure.

DAttention operates by first identifying critical regions in the feature map through multiple sets of sampling points learned from the query. Bilinear interpolation is then used to sample features from the feature map, after which the sampled features undergo key–value projection to yield deformed key–value pairs. Standard multi-head attention is subsequently applied, where the queries gather features from these deformed values. The underlying structure is illustrated in Figure 4. The input feature map $x\in R^{H\times W\times C}$ generates a uniformly spaced grid of reference points $p\in R^{H_{G}\times W_{G}\times 2}$, where grid dimensions $H_{G}=H/r$ and $W_{G}=W/r$ are determined by the downsampling factor *r*. These reference points are arranged as normalized coordinates $\left[-1,+1\right]$ relative to the grid dimensions, where $\left(-1,-1\right)$ indicates the top-left corner and $\left(+1,+1\right)$ represents the bottom-right corner.

To determine offsets for each reference point, the feature map is projected onto a query feature, denoted as $q=xW_{q}$, and then passed through a lightweight network $\theta_{offset}\left(\cdot \right)$, producing offset values $\Delta p=\theta_{offset}\left(q\right)$. These offsets modify the initial grid points, and the sampling function ϕ(·;·) combines the input features, reference points, and offsets to generate the transformed feature $\tilde{x}$. The computation is described by the following expressions:

(4) $$q=xW_{q},\tilde{k}=\tilde{x}W_{k},\tilde{v}=\tilde{x}W_{v}$$

(5) $$\Delta p=\theta_{offset}\left(q\right),\tilde{x}=\phi \left(x;p+\Delta p\right)$$

In these equations, $\tilde{k}$ and $\tilde{v}$ are the key and value matrices derived from the position-sampled features of the offset reference point. The sampling function ϕ(·;·) uses bilinear interpolation, as defined by Equation (6). Here, $\left(r_{x},r_{y}\right)$ indexes all positions in $z\in R^{H\times W\times C}$, while $\left(p_{x},p_{y}\right)$ represents the offset coordinate of reference point p+Δp:

(6) $$\phi \left(z;\left(p_{x}+p_{y}\right)\right)=\sum_{\left(r_{x},r_{y}\right)}g\left(p_{x},r_{x}\right)g\left(p_{y},r_{y}\right)z\left[r_{y},r_{x},:\right],$$

where

(7) g(a,b)=max(0,1−|a−b|)

Then, using multi-head attention and combining it with the relative position offset matrix *R* to output features, the following is obtained:

(8) $$z^{\left(m\right)}=\sigma \left(\frac{q^{\left(m\right)}{\tilde{k}}^{(m)T}}{\sqrt{d}}+\phi \left(\hat{B};R\right)\right){\tilde{v}}^{\left(m\right)}$$

### 3.5. Focaler-IoU Loss Function

Boundary box regression plays a crucial role in the field of object detection, and the localization accuracy of object detection largely depends on the loss function of boundary box regression. There is a widespread problem of sample imbalance in the perspective of drone aerial photography, where difficult samples such as pedestrians and bicycles contribute the majority of gradients, limiting the regression of bounding boxes. Based on the problem of imbalanced training samples, the Focaler-IoU [17] method is used to focus on the distribution of difficult and easy samples, and the linear interval mapping method is used to reconstruct the IoU loss. It applies a linear interval mapping to adjust the IoU loss, thereby making sample focus more adaptive. The boundary box regression loss is improved, and the formula is as follows:

(9) $$IoU^{focaler}=\left\{\begin{array}{c} 0,IoU\ll d\\ \frac{IoU-d}{u-d},d\ll IoU\ll u\\ 1,IoU\gg u \end{array}\right.$$

By adjusting the values of *u* and *d*, Focaler-IoU can be made more flexible on different samples. The definition of loss is as follows:

(10) $$L_{Focaler-Iou}=1-IoU^{Focaler}$$

After applying the Focaler-IoU loss to the boundary box regression loss based on EIoU, the contribution of high-quality samples is improved while suppressing the contribution of low-quality samples. The formula is defined as follows:

(11) $$L_{Focaler-EIou}=L_{EIoU}+IoU-IoU^{Focaler}$$

Traditional IoU-based losses (e.g., GIoU [20], DIoU, and CIoU [21]) improve localization accuracy by measuring the geometric overlap between predicted and ground-truth boxes, but they lack a mechanism to focus on sample difficulty. Focaler-IoU, on the other hand, incorporates the weight adjustment idea from Focal Loss, enabling the model to focus on bounding box regression for difficult or easy samples, thus optimizing localization performance more effectively.

### 3.6. DV-DETR Model

**Figure 5:**
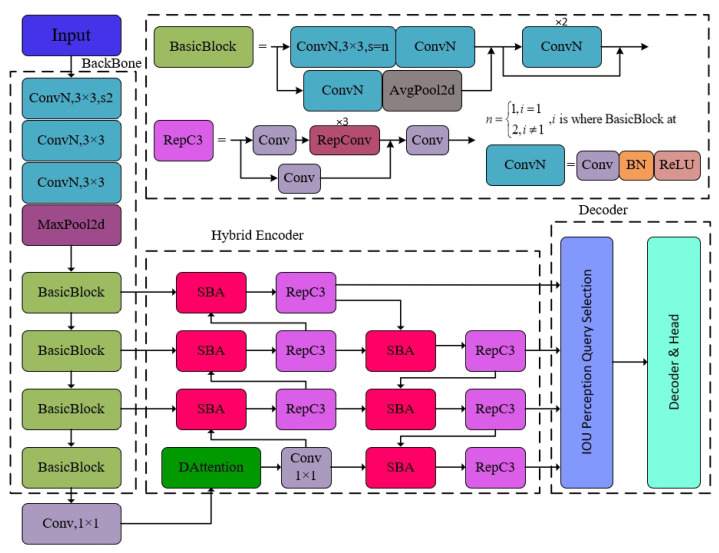
DV-DETR model structure.

To address the issue of insufficient detection performance in the RT-DETR model, recalibration attention units and reparameterization modules are introduced into the hybrid encoder of the neck network to enhance the perception of position information in small object detection and improve the learning efficiency of feature representation; at the fusion of internal scale features, the deformable attention mechanism is used to replace the baseline AIFI module in order to better aggregate the features that need attention. The final optimized object detection model DV-DETR from the perspective of unmanned aerial vehicles is shown in Figure 5.

## 4. Results

### 4.1. Dataset Introduction

To evaluate the effectiveness and applicability of our approach for small object detection, we performed experiments using the VisDrone2019 dataset, provided by the Machine Learning and Data Mining Laboratory at Tianjin University [22]. This comprehensive benchmark dataset contains 288 video clips, totaling 261,908 frames, along with 10,209 static images. This dataset includes imagery captured across 14 cities, covering a wide range of environments such as urban and rural areas, bright and dim lighting conditions, and various weather scenarios. This dataset encompasses both sparse and densely populated scenes, and is one of the largest, widest coverage, and most diverse image datasets in China’s drone aerial photography field.

The dataset defines 10 categories of detection targets, such as pedestrians, vehicles, bicycles, and more, specifically targeting small and densely packed objects, ideal for small object detection research. The target categories are defined as pedestrians, people, bicycles, cars, vans, trucks, tricycles, awning-tricycles, buses, and motorcycles. Many annotations, especially for pedestrians and distant objects, involve very small, densely clustered targets.

The dataset was divided into three subsets: 6471 images for training, 548 images for validation, and 3190 images for testing, each with a resolution of 1333 × 800. In the training set, each image was annotated with an average of 53 targets, while in the test set, each image had an average of 71 targets. These targets often exhibited varying degrees of partial occlusion in the image.

### 4.2. Experimental Environment

All network structure improvements and model training experiments were conducted in the same computer environment and configuration, as shown in Table 2.

The parameters for experimental network training are shown in Table 3, and the first 300 features were selected to initialize the decoder’s object query.

### 4.3. Model Evaluation Indicators

To verify the detection performance of the proposed model, we selected commonly used evaluation metrics in object detection tasks: precision (P), recall (R), and mean average precision (mAP). The detailed formulas for these metrics are listed below:

(12) $$P=\frac{TP}{TP+FP}$$

(13) $$R=\frac{TP}{TP+FN}$$

(14) $$AP=\int_{0}^{1}P\left(R\right)dR$$

(15) $$mAP=\frac{\sum_{i=0}^{n}AP\left(i\right)}{n}$$

In Equations (12)–(15), precision (P) refers to the proportion of correctly predicted targets among all predicted targets, while recall (R) represents the proportion of correctly predicted targets among all targets. True positive (TP) refers to the positive cases of successful prediction, false positive (FP) refers to the negative cases that are not detected, false negative (FN) refers to the positive cases that are misclassified by the model, average precision (AP) refers to the detection accuracy of each category, and n refers to the total number of categories. Additionally, parameters such as model size (number of parameters), detection speed (FPS), and computational complexity (GPLOPs) were chosen to measure the inference performance of the model.

### 4.4. Analysis of Experimental Results

#### 4.4.1. Performance Evaluation

**Figure 6:**
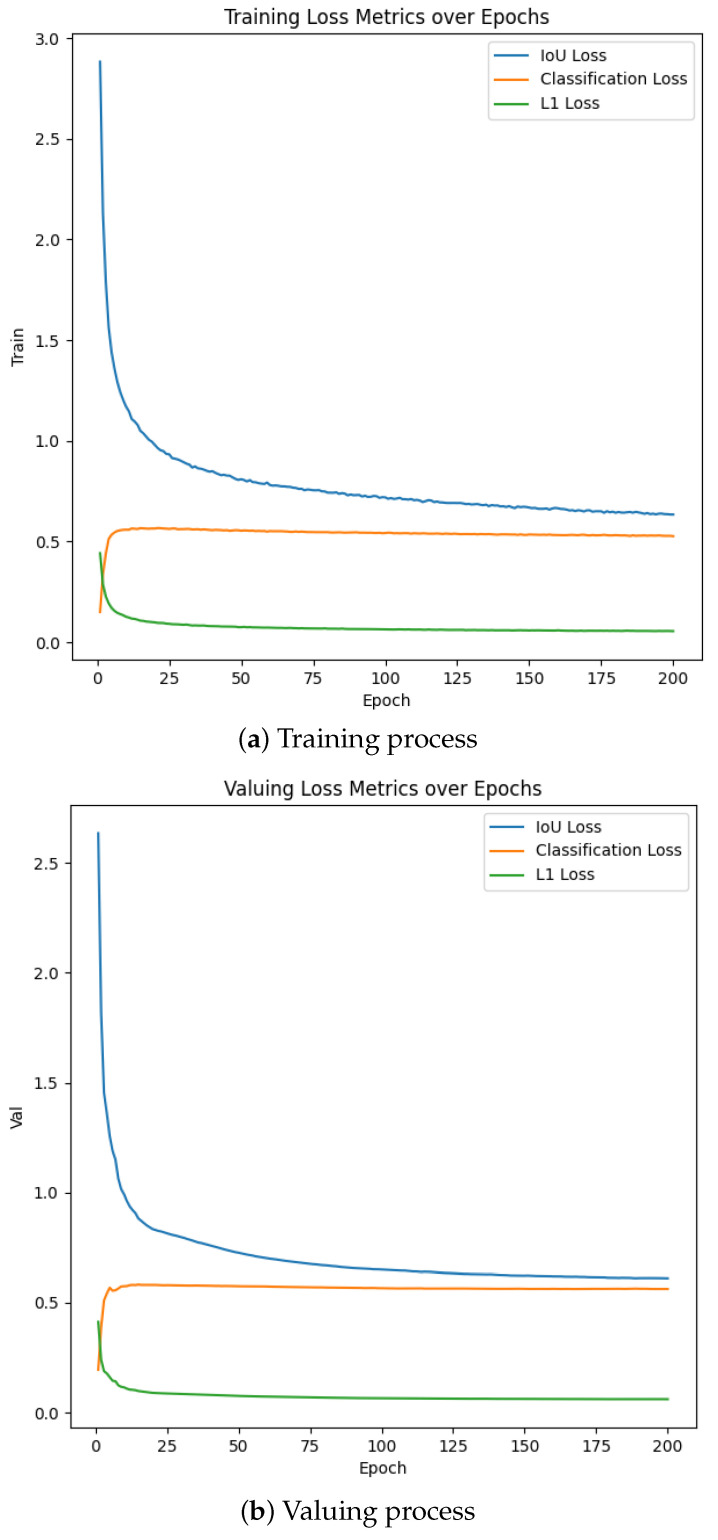
Curves in the loss function of the DV-DETR.

The variation curves of IoU loss, classification loss, and L1 loss during the training process of the improved model are shown in Figure 6. On both the training and validation sets, the IoU loss shows a rapid decreasing trend and tends to stabilize after the initial 25 training cycles. This indicates that the model quickly achieved high performance in spatial positioning accuracy and made relatively accurate predictions for bounding boxes. The decreasing trend of classification loss on the training and validation sets is relatively gentle, indicating that the classification task was more complex. Nevertheless, the loss gradually stabilizes after sufficient training epochs, indicating that the model gradually adapted to learning to distinguish between different categories of targets. The L1 loss shows a continuous decreasing trend on both the training and validation sets, reflecting the model’s continuous improvement in finely adjusting the predicted position. The stable decrease in L1 loss also indicates that the model’s ability to handle position errors gradually increased. In the end, all three types of losses tended to stabilize, indicating that the improved model proposed in this paper is effective and has a good learning effect. Overall, the loss curves on the training and validation sets show a consistent trend, indicating that the model did not experience overfitting. The performance of the model on the validation set was similar to that on the training set, indicating its good generalization ability.

Then, we present a comparative analysis of the DV-DETR model against several state-of-the-art models focused on small object detection in drone imagery. The selected models for comparison include Fast R-CNN [23], YOLOv5s, YOLOv7-t, DA-YOLO v5 [24], and RT-DETR [11]. These models were chosen due to their prominence in the field and their demonstrated effectiveness in detecting small objects in various scenarios. The experimental results are shown in Table 4. It shows that DV-DETR has better accuracy than RT-DETR in all classifications. The categories of pedestrians, people, bicycles, cars, vans, trucks, tricycles, awning-tricycles, buses, and motorcycles increased by 3.5%, 2.5%, 5.1%, 1.7%, 3.8%, 5.3%, 3.7%, 5.6%, 11.2%, and 3.7%, respectively. When IoU is 0.5, the mAP value of DV-DETRD is 50.2%, higher than RT-DETR [11] and better than all other networks. Especially in the classes of pedestrians, people, bicycles, tricycles, awning-tricycles, and motorcycles, significant AP improvements were achieved, with rates of 58.3%, 49.4%, 25.7%, 36.5%, 20.1%, and 61.2%, respectively. The mAP value of DV-DETR is 50.2%, which is 28.5%, 24%, 13.1%, 12.41%, and 4.6% higher than Fast R-CNN [23], YOLOv5s, YOLOv7-t, DA-YOLO v5 [24], and RT-DETR [11], respectively. From the experimental data, it can be concluded that the optimized network structure has a significant improvement in detecting small targets, and is capable of meeting the special needs of object detection tasks in drone aerial images.

#### 4.4.2. Ablation Experiment

In order to verify the improvement of model detection performance by various improvement schemes proposed in this article, six ablation experiments were conducted on the test set, with the same hyperparameters set for each experiment and the same training strategy used. The experimental results are shown in Table 5 and Table 6.

From Table 5, it can be seen that after replacing the RT-DETR backbone network with ResNet18 for feature extraction, the recall rate, mAP@0.5, and mAP@0.5:0.95 of ResNet18 decreased by 2.714, 2.982, and 1.9 percentage points compared to the baseline model. However, Table 5 shows that FPS increased by 64.1, and GFLOPs and parameter count decreased by 51.5 and 14.557M, respectively. This indicates that ResNet18, as a lightweight backbone network, has slightly inferior feature extraction capabilities compared to the baseline model, but significantly reduces the overall network parameter count, which can meet the requirements of real-time detection. After adopting the SBA module and adjusting the neck network structure, the precision, recall rate, and mAP@0.5 decreased by 1.94, 3.297, and 4.031 percentage points compared to experiment 2, which indicates that the SBA module can greatly improve the model’s detection performance for small targets and significantly increase the average accuracy by fusing high-resolution and low-resolution features to better handle multi-scale information. After adopting the DAttention module, the precision and recall rate increased by 0.333 and 0.325 percentage points, respectively, compared to experiment 3, indicating that this mechanism can model the relationship between markers in complex and changing scenarios, thus paying more attention to small targets. After using Focaler-IoU loss, the precision and mAP@0.5:0.95 increased by 0.426 and 0.1 percentage points compared to experiment 3, indicating that Focaler-IoU pays attention to the distribution of difficult and easy samples, thereby improving the contribution of high-quality samples while suppressing the contribution of low-quality samples. The precision, recall, mAP@0.5, and mAP@0.5:0.95 of the final improved DV-DETR model increased by 0.284, 1.102, 1.661, and 1.7 percentage points compared to the baseline model, respectively, indicating that DV-DETR is more efficient in fine feature extraction and high/low-resolution feature fusion, and the drone has stronger real-time small target detection capabilities.

Table 6 compares the inference performance of the improved model against others. The mechanism of bidirectional feature fusion after adding the SBA module can preserve more feature information, resulting in an increase in computational complexity and parameter quantity of the model, and a slight decrease in detection speed. After introducing the DAttention module, it focused more specifically on related feature information, with slightly increased computational complexity and parameter count, and almost no change in detection speed. Compared with the original model, the improved DV-DETR model showed varying degrees of improvement in various detection performance evaluation indicators, and reduced computational complexity and parameter count. It performed better in real-time detection tasks for small targets in UAV aerial photography.

## 5. Conclusions

To address the challenge of low real-time detection accuracy of small targets in UAV aerial photography with high viewing angles and dynamic scenarios, this paper presents an enhanced DV-DETR model based on RT-DETR. By substituting the backbone network with ResNet18, the model achieves better extraction of multi-level image features, which improves small target detection accuracy and localization precision, while also significantly reducing model parameters. In the feature fusion process, the recalibration attention unit, which aggregates refined boundary and the semantic information to better calibrate small object positions, and the reparameterization module, which uses a multi-branch structure during training to optimize multi-scale feature extraction and consolidates to a single-branch structure during inference, are incorporated to enable effective bidirectional fusion of high- and low-resolution features, enhancing the model’s ability to handle multi-scale targets. Additionally, the DAttention mechanism models relationships between target labels to better aggregate important features. Finally, the Focaler-IoU loss function is utilized to adjust weights for positive and negative samples, optimizing the model’s focus on challenging samples and enhancing its small target recognition performance. Experimental results indicate that DV-DETR substantially boosts the effectiveness of drone-based monitoring systems, particularly in complex urban environments with crowded scenes. While the proposed DV-DETR model exhibits promising performance, further refinement and extensive field testing are required to validate its robustness in diverse operational conditions. Future testing in a variety of environments, including different weather conditions, longer observation periods, and more diverse datasets, would facilitate comprehensive validation and adaptation of the model for practical applications.

## Figures and Tables

**Table 1:** Performance comparison of different backbone networks.

Backbone Network	Parameters/M	GFLOPs	Mean Average Precision mAP/%
ResNet18	17.657	53.4	45.407
SwinTransformer	36.620	98.1	46.579
HGNet	32.827	108.3	48.389

**Table 2:** Experimental environment.

Configurations	Parameters
OS	Ubuntu 20.04
CPU	Intel Xeon E5-2680
GPU	NVIDIA GeForce RTX 3090 24 GB
CUDA Version	CUDA 12.1
Memory	64 GB
Deep Learning Framework	Pytorch 2.2.0

**Table 3:** Training parameters.

Name	Parameters
Epoch	200
Batch Size	8
Input Size	640 × 640
Optimizer	AdamW
Initial Learning Rate	0.0001
Weight Decay Coefficient	0.0001

**Table 4:** Detection results of each model (%). (The bold data in the table indicate the best results).

Model	Pedestrians	People	Bicycles	Cars	Vans	Trucks	Tricycles	Awning-Tricycles	Buses	Motorcycles	mAP@0.5
Fast R-CNN [23]	21.4	15.6	6.7	51.7	29.5	19	13.1	7.7	31.4	20.7	21.7
YOLOv5s	22.6	20.6	14.6	59.7	24	21.3	20.1	17.4	37.9	23.7	26.2
YOLOv7-t	41.5	37.5	11.4	77.9	39.8	32.8	23.7	12	48.1	46.7	37.1
DA-YOLO v5 [24]	47.1	37.9	14.2	78.3	37.4	32.3	23.5	15.6	47.2	44.4	37.79
RT-DETR [11]	54.8	46.9	20.6	85.1	49.5	35.9	32.8	14.5	58.1	57.5	45.6
**DV-DETR**	**58.3**	**49.4**	**25.7**	**86.8**	**53.3**	**41.2**	**36.5**	**20.1**	**69.3**	**61.2**	**50.2**

**Table 5:** Ablation experimental results (%).

Number	Models	P	R	mAP@0.5	mAP@0.5:0.95
Exp1	RT-DETR (Baseline)	62.147	46.657	48.389	29.7
Exp2	+ ResNet18	59.293	43.943	45.407	27.8
Exp3	+ ResNet18 + SBA Module	61.233	47.240	49.438	30.9
Exp4	+ ResNet18 + SBA Module + DAttention	61.566	47.565	49.496	31.1
Exp5	+ ResNet18 + SBA Module + Focaler-IoU	61.559	47.314	49.462	31.0
Exp6	+ ResNet18 + SBA Module + DAttention + Focaler-IoU	62.431	47.759	50.050	31.4

**Table 6:** Comparative results of model inference performance in ablation experiments.

Number	Models	FPS	GFLOPs	Params/10^6^
Exp1	RT-DETR (Baseline)	75.0	103.5	32.004
Exp2	+ ResNet18	139.1 (+64.1)	52.0 (−51.5)	17.447 (−14.557)
Exp3	+ ResNet18 + SBA Module	92.0 (+17.0)	84.4 (−19.1)	19.498 (−12.506)
Exp4	+ ResNet18 + SBA Module + DAttention	89.6 (+14.6)	84.6 (−18.9)	19.501 (−12.503)
Exp5	+ ResNet18 + SBA Module + Focaler-IoU	90.3 (+15.3)	84.4 (−19.1)	19.498 (−12.506)
Exp6	+ ResNet18 + SBA Module + DAttention + Focaler-IoU	90.0 (+15.0)	84.6 (−18.9)	19.501 (−12.503)

## Data Availability

The raw data supporting the conclusions of this article will be made available by the authors on request.
